# SUBJECTIVE COGNITIVE DECLINE AND RISK OF MORTALITY AMONG BEREAVED PARENTS IN OLD AGE

**DOI:** 10.1093/geroni/igae098.3727

**Published:** 2024-12-31

**Authors:** Jieun Song, Seung Eun Cha, Carol Ryff

**Affiliations:** University of Wisconsin-Madison, Madison, Wisconsin, United States; University of Wisconsin-Madison, Madison, Wisconsin, United States; University of Wisconsin-Madison, Madison, Wisconsin, United States

## Abstract

The death of a child is a highly traumatic event that may impact the subsequent health of parents. Prior research shows greater risk of early deaths in bereaved parents relative to non-bereaved parents. This study examined whether the associations between the death of a child and parents’ mortality risk are mediated by subjective cognitive decline (SCD), known to be predictive of mortality and Alzheimer’s disease, contingent on parents’ age. The analytic sample consists of participants in Midlife in the U.S. (MIDUS) waves II and III (2004-2005, 2013-2014, respectively); parents who experienced the death of a child prior to MIDUS II (n=341) and non-bereaved parents who did not experience the death of a child (n=2,219). SCD was assessed at MIDUS III and mortality data were obtained via National Death Index 2022 files. Results of moderated mediation analysis revealed significant mediation effects of SCD between death of a child and parental mortality risks over time among parents who were aged 65 and older at SCD assessment, but not among parents who were younger than 65. Bereaved parents who reported a decline in memory at age 65 or older had greater risks of mortality over 8-9 years relative to their counterparts. Findings suggest that increased risks of mortality in bereaved parents during old age can be detected by a relatively simple assessment of SCD, which can be utilized to screen vulnerable bereaved parent groups who may benefit from interventions to mitigate detrimental impacts of bereavement on mortality risks.

